# Supplementary material for: Resolving the full spectrum of human genome variation using Linked-Reads
Source: Genome Res. 2019 Apr;29(4):635–45. doi: 10.1101/gr.234443.118 (PMC6442396; doi:10.1101/gr.234443.118)
Supplement: Supplemental Material [file supp_gr.234443.118_Supplemental_longranger-2.2.zip › longranger-2.2.tar.bz2!/lr2.2-public/lib/stan/cmdstan/stan/doc/api/html/agrad_2rev_2matrix_2mdivide__left_8hpp_source.html]

Stan: src/stan/agrad/rev/matrix/mdivide\_left.hpp Source File


|  |  |
| --- | --- |
|  | Stan  2.5.0  probability, sampling & optimization |


- Main Page
- Related Pages
- Namespaces
- Classes
- Files

- File List
- File Members

All Classes Namespaces Files Functions Variables Typedefs Enumerations Enumerator Friends Macros Pages

- src
- stan
- agrad
- rev
- matrix

mdivide\_left.hpp

Go to the documentation of this file.

1 #ifndef STAN\_\_AGRAD\_\_REV\_\_MATRIX\_\_COLUMNS\_MDIVIDE\_LEFT\_HPP

2 #define STAN\_\_AGRAD\_\_REV\_\_MATRIX\_\_COLUMNS\_MDIVIDE\_LEFT\_HPP

3

4 #include <vector>

5 #include <stan/math/matrix/Eigen.hpp>

6 #include <stan/math/matrix/typedefs.hpp>

7 #include <stan/math/error\_handling/matrix/check\_multiplicable.hpp>

8 #include <stan/agrad/rev/var.hpp>

9 #include <stan/agrad/rev/matrix/typedefs.hpp>

10 #include <stan/math/error\_handling/matrix/check\_square.hpp>

11

12 namespace stan {

13  namespace agrad {

14

15  namespace {

16  template <int R1,int C1,int R2,int C2>

17  class mdivide\_left\_vv\_vari : public vari {

18  public:

19  int M\_; // A.rows() = A.cols() = B.rows()

20  int N\_; // B.cols()

21  double\* A\_;

22  double\* C\_;

23  vari\*\* \_variRefA;

24  vari\*\* \_variRefB;

25  vari\*\* \_variRefC;

26

27  mdivide\_left\_vv\_vari(const Eigen::Matrix<var,R1,C1> &A,

28  const Eigen::Matrix<var,R2,C2> &B)

29  : vari(0.0),

30  M\_(A.rows()),

31  N\_(B.cols()),

32  A\_((double\*)stan::agrad::memalloc\_.alloc(sizeof(double)

33  \* A.rows() \* A.cols())),

34  C\_((double\*)stan::agrad::memalloc\_.alloc(sizeof(double)

35  \* B.rows() \* B.cols())),

36  \_variRefA((vari\*\*)stan::agrad::memalloc\_.alloc(sizeof(vari\*)

37  \* A.rows() \* A.cols())),

38  \_variRefB((vari\*\*)stan::agrad::memalloc\_.alloc(sizeof(vari\*)

39  \* B.rows() \* B.cols())),

40  \_variRefC((vari\*\*)stan::agrad::memalloc\_.alloc(sizeof(vari\*)

41  \* B.rows() \* B.cols()))

42  {

43  using Eigen::Matrix;

44  using Eigen::Map;

45

46  size\_t pos = 0;

47  for (size\_type j = 0; j < M\_; j++) {

48  for (size\_type i = 0; i < M\_; i++) {

49  \_variRefA[pos] = A(i,j).vi\_;

50  A\_[pos++] = A(i,j).val();

51  }

52  }

53

54  pos = 0;

55  for (size\_type j = 0; j < N\_; j++) {

56  for (size\_type i = 0; i < M\_; i++) {

57  \_variRefB[pos] = B(i,j).vi\_;

58  C\_[pos++] = B(i,j).val();

59  }

60  }

61

62  Matrix<double,R1,C2> C(M\_,N\_);

63  C = Map<Matrix<double,R1,C2> >(C\_,M\_,N\_);

64

65  C = Map<Matrix<double,R1,C1> >(A\_,M\_,M\_)

66  .colPivHouseholderQr().solve(C);

67

68  pos = 0;

69  for (size\_type j = 0; j < N\_; j++) {

70  for (size\_type i = 0; i < M\_; i++) {

71  C\_[pos] = C(i,j);

72  \_variRefC[pos] = new vari(C\_[pos],false);

73  pos++;

74  }

75  }

76  }

77

78  virtual void chain() {

79  using Eigen::Matrix;

80  using Eigen::Map;

81  Eigen::Matrix<double,R1,C1> adjA(M\_,M\_);

82  Eigen::Matrix<double,R2,C2> adjB(M\_,N\_);

83  Eigen::Matrix<double,R1,C2> adjC(M\_,N\_);

84

85  size\_t pos = 0;

86  for (size\_type j = 0; j < adjC.cols(); j++)

87  for (size\_type i = 0; i < adjC.rows(); i++)

88  adjC(i,j) = \_variRefC[pos++]->adj\_;

89

90

91  adjB = Map<Matrix<double,R1,C1> >(A\_,M\_,M\_)

92  .transpose().colPivHouseholderQr().solve(adjC);

93  adjA.noalias() = -adjB

94  \* Map<Matrix<double,R1,C2> >(C\_,M\_,N\_).transpose();

95

96  pos = 0;

97  for (size\_type j = 0; j < adjA.cols(); j++)

98  for (size\_type i = 0; i < adjA.rows(); i++)

99  \_variRefA[pos++]->adj\_ += adjA(i,j);

100

101  pos = 0;

102  for (size\_type j = 0; j < adjB.cols(); j++)

103  for (size\_type i = 0; i < adjB.rows(); i++)

104  \_variRefB[pos++]->adj\_ += adjB(i,j);

105  }

106  };

107

108  template <int R1,int C1,int R2,int C2>

109  class mdivide\_left\_dv\_vari : public vari {

110  public:

111  int M\_; // A.rows() = A.cols() = B.rows()

112  int N\_; // B.cols()

113  double\* A\_;

114  double\* C\_;

115  vari\*\* \_variRefB;

116  vari\*\* \_variRefC;

117

118  mdivide\_left\_dv\_vari(const Eigen::Matrix<double,R1,C1> &A,

119  const Eigen::Matrix<var,R2,C2> &B)

120  : vari(0.0),

121  M\_(A.rows()),

122  N\_(B.cols()),

123  A\_((double\*)stan::agrad::memalloc\_.alloc(sizeof(double)

124  \* A.rows() \* A.cols())),

125  C\_((double\*)stan::agrad::memalloc\_.alloc(sizeof(double)

126  \* B.rows() \* B.cols())),

127  \_variRefB((vari\*\*)stan::agrad::memalloc\_.alloc(sizeof(vari\*)

128  \* B.rows() \* B.cols())),

129  \_variRefC((vari\*\*)stan::agrad::memalloc\_.alloc(sizeof(vari\*)

130  \* B.rows() \* B.cols()))

131  {

132  using Eigen::Matrix;

133  using Eigen::Map;

134

135  size\_t pos = 0;

136  for (size\_type j = 0; j < M\_; j++) {

137  for (size\_type i = 0; i < M\_; i++) {

138  A\_[pos++] = A(i,j);

139  }

140  }

141

142  pos = 0;

143  for (size\_type j = 0; j < N\_; j++) {

144  for (size\_type i = 0; i < M\_; i++) {

145  \_variRefB[pos] = B(i,j).vi\_;

146  C\_[pos++] = B(i,j).val();

147  }

148  }

149

150  Matrix<double,R1,C2> C(M\_,N\_);

151  C = Map<Matrix<double,R1,C2> >(C\_,M\_,N\_);

152

153  C = Map<Matrix<double,R1,C1> >(A\_,M\_,M\_)

154  .colPivHouseholderQr().solve(C);

155

156  pos = 0;

157  for (size\_type j = 0; j < N\_; j++) {

158  for (size\_type i = 0; i < M\_; i++) {

159  C\_[pos] = C(i,j);

160  \_variRefC[pos] = new vari(C\_[pos],false);

161  pos++;

162  }

163  }

164  }

165

166  virtual void chain() {

167  using Eigen::Matrix;

168  using Eigen::Map;

169  Eigen::Matrix<double,R2,C2> adjB(M\_,N\_);

170  Eigen::Matrix<double,R1,C2> adjC(M\_,N\_);

171

172  size\_t pos = 0;

173  for (size\_type j = 0; j < adjC.cols(); j++)

174  for (size\_type i = 0; i < adjC.rows(); i++)

175  adjC(i,j) = \_variRefC[pos++]->adj\_;

176

177  adjB = Map<Matrix<double,R1,C1> >(A\_,M\_,M\_)

178  .transpose().colPivHouseholderQr().solve(adjC);

179

180  pos = 0;

181  for (size\_type j = 0; j < adjB.cols(); j++)

182  for (size\_type i = 0; i < adjB.rows(); i++)

183  \_variRefB[pos++]->adj\_ += adjB(i,j);

184  }

185  };

186

187  template <int R1,int C1,int R2,int C2>

188  class mdivide\_left\_vd\_vari : public vari {

189  public:

190  int M\_; // A.rows() = A.cols() = B.rows()

191  int N\_; // B.cols()

192  double\* A\_;

193  double\* C\_;

194  vari\*\* \_variRefA;

195  vari\*\* \_variRefC;

196

197  mdivide\_left\_vd\_vari(const Eigen::Matrix<var,R1,C1> &A,

198  const Eigen::Matrix<double,R2,C2> &B)

199  : vari(0.0),

200  M\_(A.rows()),

201  N\_(B.cols()),

202  A\_((double\*)stan::agrad::memalloc\_.alloc(sizeof(double)

203  \* A.rows() \* A.cols())),

204  C\_((double\*)stan::agrad::memalloc\_.alloc(sizeof(double)

205  \* B.rows() \* B.cols())),

206  \_variRefA((vari\*\*)stan::agrad::memalloc\_.alloc(sizeof(vari\*)

207  \* A.rows() \* A.cols())),

208  \_variRefC((vari\*\*)stan::agrad::memalloc\_.alloc(sizeof(vari\*)

209  \* B.rows() \* B.cols()))

210  {

211  using Eigen::Matrix;

212  using Eigen::Map;

213

214  size\_t pos = 0;

215  for (size\_type j = 0; j < M\_; j++) {

216  for (size\_type i = 0; i < M\_; i++) {

217  \_variRefA[pos] = A(i,j).vi\_;

218  A\_[pos++] = A(i,j).val();

219  }

220  }

221

222  Matrix<double,R1,C2> C(M\_,N\_);

223  C = Map<Matrix<double,R1,C1> >(A\_,M\_,M\_)

224  .colPivHouseholderQr().solve(B);

225

226  pos = 0;

227  for (size\_type j = 0; j < N\_; j++) {

228  for (size\_type i = 0; i < M\_; i++) {

229  C\_[pos] = C(i,j);

230  \_variRefC[pos] = new vari(C\_[pos],false);

231  pos++;

232  }

233  }

234  }

235

236  virtual void chain() {

237  using Eigen::Matrix;

238  using Eigen::Map;

239  Eigen::Matrix<double,R1,C1> adjA(M\_,M\_);

240  Eigen::Matrix<double,R1,C2> adjC(M\_,N\_);

241

242  size\_t pos = 0;

243  for (size\_type j = 0; j < adjC.cols(); j++)

244  for (size\_type i = 0; i < adjC.rows(); i++)

245  adjC(i,j) = \_variRefC[pos++]->adj\_;

246

247  // FIXME: add .noalias() to LHS

248  adjA = -Map<Matrix<double,R1,C1> >(A\_,M\_,M\_)

249  .transpose()

250  .colPivHouseholderQr()

251  .solve(adjC\*Map<Matrix<double,R1,C2> >(C\_,M\_,N\_).transpose());

252

253  pos = 0;

254  for (size\_type j = 0; j < adjA.cols(); j++)

255  for (size\_type i = 0; i < adjA.rows(); i++)

256  \_variRefA[pos++]->adj\_ += adjA(i,j);

257  }

258  };

259  }

260

261  template <int R1,int C1,int R2,int C2>

262  inline

263  Eigen::Matrix<var,R1,C2>

264  mdivide\_left(const Eigen::Matrix<var,R1,C1> &A,

265  const Eigen::Matrix<var,R2,C2> &b) {

266  Eigen::Matrix<var,R1,C2> res(b.rows(),b.cols());

267

268  stan::math::check\_square("mdivide\_left(%1%)",A,"A",(double\*)0);

269  stan::math::check\_multiplicable("mdivide\_left(%1%)",A,"A",

270  b,"b",(double\*)0);

271

272  // NOTE: this is not a memory leak, this vari is used in the

273  // expression graph to evaluate the adjoint, but is not needed

274  // for the returned matrix. Memory will be cleaned up with the arena allocator.

275  mdivide\_left\_vv\_vari<R1,C1,R2,C2> \*baseVari = new mdivide\_left\_vv\_vari<R1,C1,R2,C2>(A,b);

276

277  size\_t pos = 0;

278  for (size\_type j = 0; j < res.cols(); j++)

279  for (size\_type i = 0; i < res.rows(); i++)

280  res(i,j).vi\_ = baseVari->\_variRefC[pos++];

281

282  return res;

283  }

284

285  template <int R1,int C1,int R2,int C2>

286  inline

287  Eigen::Matrix<var,R1,C2>

288  mdivide\_left(const Eigen::Matrix<var,R1,C1> &A,

289  const Eigen::Matrix<double,R2,C2> &b) {

290  Eigen::Matrix<var,R1,C2> res(b.rows(),b.cols());

291

292  stan::math::check\_square("mdivide\_left(%1%)",A,"A",(double\*)0);

293  stan::math::check\_multiplicable("mdivide\_left(%1%)",A,"A",

294  b,"b",(double\*)0);

295

296  // NOTE: this is not a memory leak, this vari is used in the

297  // expression graph to evaluate the adjoint, but is not needed

298  // for the returned matrix. Memory will be cleaned up with the arena allocator.

299  mdivide\_left\_vd\_vari<R1,C1,R2,C2> \*baseVari = new mdivide\_left\_vd\_vari<R1,C1,R2,C2>(A,b);

300

301  size\_t pos = 0;

302  for (size\_type j = 0; j < res.cols(); j++)

303  for (size\_type i = 0; i < res.rows(); i++)

304  res(i,j).vi\_ = baseVari->\_variRefC[pos++];

305

306  return res;

307  }

308

309  template <int R1,int C1,int R2,int C2>

310  inline

311  Eigen::Matrix<var,R1,C2>

312  mdivide\_left(const Eigen::Matrix<double,R1,C1> &A,

313  const Eigen::Matrix<var,R2,C2> &b) {

314  Eigen::Matrix<var,R1,C2> res(b.rows(),b.cols());

315

316  stan::math::check\_square("mdivide\_left(%1%)",A,"A",(double\*)0);

317  stan::math::check\_multiplicable("mdivide\_left(%1%)",A,"A",

318  b,"b",(double\*)0);

319

320  // NOTE: this is not a memory leak, this vari is used in the

321  // expression graph to evaluate the adjoint, but is not needed

322  // for the returned matrix. Memory will be cleaned up with the arena allocator.

323  mdivide\_left\_dv\_vari<R1,C1,R2,C2> \*baseVari = new mdivide\_left\_dv\_vari<R1,C1,R2,C2>(A,b);

324

325  size\_t pos = 0;

326  for (size\_type j = 0; j < res.cols(); j++)

327  for (size\_type i = 0; i < res.rows(); i++)

328  res(i,j).vi\_ = baseVari->\_variRefC[pos++];

329

330  return res;

331  }

332

333  }

334 }

335 #endif

N\_

int N\_

**Definition:** mdivide\_left.hpp:20

check\_multiplicable.hpp

typedefs.hpp

typedefs.hpp

A\_

double \* A\_

**Definition:** mdivide\_left.hpp:21

stan::agrad::mdivide\_left

Eigen::Matrix< fvar< T >, R1, C2 > mdivide\_left(const Eigen::Matrix< fvar< T >, R1, C1 > &A, const Eigen::Matrix< fvar< T >, R2, C2 > &b)

**Definition:** mdivide\_left.hpp:25

stan::agrad::memalloc\_

memory::stack\_alloc memalloc\_

**Definition:** var\_stack.cpp:16

\_variRefA

vari \*\* \_variRefA

**Definition:** mdivide\_left.hpp:23

stan::math::check\_multiplicable

bool check\_multiplicable(const char \*function, const T1 &y1, const char \*name1, const T2 &y2, const char \*name2, T\_result \*result)

**Definition:** check\_multiplicable.hpp:15

stan::math::transpose

Eigen::Matrix< T, C, R > transpose(const Eigen::Matrix< T, R, C > &m)

**Definition:** transpose.hpp:12

\_variRefB

vari \*\* \_variRefB

**Definition:** mdivide\_left.hpp:24

stan::agrad::size\_type

Eigen::Matrix< double, Eigen::Dynamic, Eigen::Dynamic >::Index size\_type

**Definition:** typedefs.hpp:14

stan::math::rows

size\_t rows(const Eigen::Matrix< T, R, C > &m)

**Definition:** rows.hpp:12

C\_

double \* C\_

**Definition:** mdivide\_left.hpp:22

var.hpp

stan::math::cols

size\_t cols(const Eigen::Matrix< T, R, C > &m)

**Definition:** cols.hpp:12

Eigen.hpp

\_variRefC

vari \*\* \_variRefC

**Definition:** mdivide\_left.hpp:25

stan::math::check\_square

bool check\_square(const char \*function, const Eigen::Matrix< T\_y, Eigen::Dynamic, Eigen::Dynamic > &y, const char \*name, T\_result \*result)

Return true if the specified matrix is square.

**Definition:** check\_square.hpp:24

M\_

int M\_

**Definition:** mdivide\_left.hpp:19

check\_square.hpp


---

[ Stan Home Page ]

*© 2011–2014,
Stan Development Team.*
